# A non-randomised experimental feasibility study into the immediate effect of three different spinal manipulative protocols on kicking speed performance in soccer players

**DOI:** 10.1186/s12998-014-0046-3

**Published:** 2015-01-13

**Authors:** Kyle Colin Deutschmann, Andrew Douglas Jones, Charmaine Maria Korporaal

**Affiliations:** M.Tech:Chiropractic, Durban, South Africa; M.Dip:Chiropractic, MMedSci (Sports Science), CCSP, Durban, South Africa; Department of Chiropractic and Somatology, Chiropractic Programme, M.Tech:Chiropractic, CCFC, CCSP, ICSSD, Durban University of Technology, Durban, South Africa

**Keywords:** Chiropractic, Manipulation, Athletic performance, Soccer

## Abstract

**Background:**

The most utilized soccer kicking method is the instep kicking technique. Decreased motion in spinal joint segments results in adverse biomechanical changes within in the kinematic chain. These changes may be linked to a negative impact on soccer performance. This study tested the immediate effect of lumbar spine and sacroiliac manipulation alone and in combination on the kicking speed of uninjured soccer players.

**Methods:**

This 2010 prospective, pre-post experimental, single-blinded (subject) required forty asymptomatic soccer players, from regional premier league teams, who were purposively allocated to one of four groups (based on the evaluation of the players by two blinded motion palpators). Segment dysfunction was either localized to the lumbar spine (Group 1), sacroiliac joint (Group 2), the lumbar spine and sacroiliac joint (Group 3) or not present in the sham laser group (Group 4). All players underwent a standardized warm-up before the pre-measurements. Manipulative intervention followed after which post-measurements were completed. Measurement outcomes included range of motion changes (digital inclinometer); kicking speed (Speed Trac™ Speed Sport Radar) and the subjects’ perception of a change in kicking speed. SPSS version 15.0 was used to analyse the data, with repeated measures ANOVA and a *p*-value <0.05 (CI 95%).

**Results:**

Lumbar spine manipulation resulted in significant range of motion increases in left and right rotation. Sacroiliac manipulation resulted in no significant changes in the lumbar range of motion. Combination manipulative interventions resulted in significant range of motion increases in lumbar extension, right rotation and right SI joint flexion. There was a significant increase in kicking speed post intervention for all three manipulative intervention groups (when compared to sham). A significant correlation was seen between Likert based-scale subjects’ perception of change in kicking speed post intervention and the objective results obtained.

**Conclusions:**

This pilot study showed that lumbar spine manipulation combined with SI joint manipulation, resulted in an effective intervention for short-term increases in kicking speed/performance. However, the lack of an *a priori* analysis, a larger sample size and an unblinded outcome measures assessor requires that this study be repeated, addressing these concerns and for these outcomes to be validated.

## Introduction

The instep kicking technique is the most commonly used kicking technique in soccer, which allows the development of an optimum kicking speed [1-3]. This kicking technique requires that the power is generated through the co-ordinated effort of the muscles and the motion of all the joints involved (viz. lumbar spine, sacroiliac joint, hip, knee and foot and ankle) [4,5]. Thus, this kicking technique’s biomechanics are seen as a segmented motion pattern sequence which initiates from the at the spine and moves distally down the open biomechanical chain [4-7]. As, the lumbar spine and sacroiliac joint are both proximal parts of this biomechanical chain, they form the basis for motion which follows the open chain movement pattern, and thus initiate the forward motion during kicking [2,5]. Thus musculoskeletal co-ordination forms the basis for the kicking action and closely controls the compression forces being transferred towards the spine, stabilising and keeping the upper body balanced and upright, whilst transmitting the requires forces down the kinematic chain [8].

To achieve the above, the player’s approach or backswing phase of the kicking technique requires that the lumbar spine rotates posteriorly and extends allowing the trunk to rotate towards the kicking leg [2,9,10]. At the end of the swing limb loading phase the lumbar spine is rotated and extended, in accordance with soccer technique, in order to appropriately load the thoraco-lumbar fascia for recoil and wind up prior to the kick. This increase in musculo-ligamentous torque during in the wind up, allows for maximum distance to be achieved when striking the ball. Once, the swing phase is initiated by the trunk, the lumbar spine rotates towards the supporting leg to transfer momentum from the larger proximal segments to the distal smaller ones, in order to accelerate the kicking limb into flexion at the hip [2,9,10], as it speeds towards the ball.

At this point Cohan [11] and Gilchrist et al., [8], concur that the hip and sacroiliac musculature are required to work together to effect movement of the pelvis (for example hip joint extension causes anterior pelvic tilt and extending the SI joint). Similarly, hip flexion is associated with posterior pelvic tilt and allows the SI joint to assume a flexed position [2]. By contrast, during the foot planting phase, the SI joint is active in absorbing and controlling the force being transmitted through the body and down the biomechanical chain, as a result of the ground reactive force acting on the limb [2].

It is therefore evident that the instep soccer kick is a complex maneuver, on which the outcome of a soccer game depends [12,13]. Thus, players are expected to perform this “routine action” at their maximum potential every time they kick the ball to score. This co-ordination of this components of this complex maneuver impacts on the kicking speed [direct result of a summation of forces created by the musculoskeletal basis of the kick and its generated momentum down the biomechanical chain] [2,4,5,8]. In addition, an increase in the distance over which the open kinematic chain can move, it is hypothesized that there will be an increase in the potential to achieve a higher foot speed at the point of impact [2,9,10,14].

This hypothesis concurs with the literature, which indicates that when immobilization or restricted motion exists within any of these joint segments, it results in adverse changes in the surrounding ligaments, tendons, muscular tissue and vascular elements [15-17]. It is through these functional impairments with a loss of tensile strength of ligaments, adhesions formation [15,16,18,19], loss of muscular or ligamentous flexibility and joint range of motion (ROM) decreases [17,19-22], that performance may be decreased . Therefore it is the opinion of several authors that improved spinal joint mobility and muscle flexibility can be achieved through the use of manipulation [15,16,22-25].

Thus the restoration of normal biomechanics and neurological input [26-29], increased flexibility and mobility of joints and surrounding tissues resulting from manipulation [23,30,31] may result in increased speed of the biomechanical chain during the kicking motion.

There is however, limited published literature on the immediate post manipulation effect of manipulation on the ROM of the low back joints in asymptomatic subjects. Therefore, this study determined whether manipulation of the lumbar spine and the sacroiliac joints increased the ROM at within these anatomical regions (measured goniometrically) and whether this was associated with changes in kicking speed and the subjective perception of the kicking ability.

## Method

### Recruitment and informed consent

On Institutional Research and Ethics Committee of the Durban University of Technology (034/10) approval of this study in 2010, the subjects were recruited after permission was received from the Highway Action Center. Players were informed of the study by the placement of advertisements at the arena and through word of mouth. In addition players in the regional premier league teams were approached by the researcher in order to request participation (convenience sampling) [32]. Subsequent interaction with the potential subjects required that the soccer players read and understood the letter of information and informed consent as approved by the IRB and agreed to participate by voluntarily signing the informed consent.

On agreement to participate the subjects were then required to undergo a clinical assessment (case history, physical and orthopedic examinations), which was administered at the Chiropractic Day Clinic, to ensure that the subjects complied with the inclusion criteria.

### Sample size and allocation

A sample size of 40, asymptomatic subjects was required for this study, resulting in ten subjects in each of four intervention groups. Due to the lack of access to national league teams, the researcher was limited to regional premier league teams. This resulted in a relatively small sample pool (approximately 75 soccer players) from which to draw subjects for this study. As a result the sample size was based on a pragmatic decision rather than a statistical evaluation of sample size (viz. *a prior* analysis).

The subjects were purposively assigned to one of four intervention groups, based on the level of the motion segment dysfunction. This was based on a standardised motion palpation protocol developed from Bergmann and Peterson [21], Schafer and Faye [33] and Bergmann, Peterson and Lawrence [34], of the lumbar spine and sacroiliac joints. This procedure was performed independently by both the researcher and the clinical supervisor [35]. The subjects were allocated to their respective group - lumbar spine (Group 1), sacroiliac joint (Group 2), the lumbar spine and sacroiliac joint (Group 3) - by those joint dysfunctions that were commonly agreed to by the researcher and the clinical supervisor. Those subjects with no joint dysfunction in the palpated joints were placed into Group 4.

### Sample characteristics

Subjects were required to be males, between the ages of 18 to 35 and had to be soccer athletes (no distinction was made with regard to player position), as all players must be able to kick and due to the small numbers that were available. Subjects were required to have clinical signs of joint dysfunction (asymptomatic, e.g. pain) [21,34] in either the lumbar spine or the sacroiliac joints or both. Exclusion criteria included subjects who presented with contraindications to spinal manipulations [21,34].

### Procedure

After subjects signed informed consent, inclusion into the study (at the initial consultation) and allocation to a group (at the data collection arena/subsequent consultation) was determined. All players were instructed through a standardized warm-up procedure prior to measurements being taken. Each player was taken through a standardized procedure required which included a warm up run around the outside of an indoor court, a seated self stretch of the hamstrings, a prone self stretch for the quadriceps femoris, a seated stretch for the adductor muscles, a supine stretch for the quadratus lumborum and a standing gastrocnemius and soleus [36].

After the completion of the warm up procedure the pre-intervention measurements were taken: lumbar (flexion, extension, lateral flexion, and rotation) and sacroiliac (flexion and extension) range of motion parameters. The player was then required to complete a maximum run-up distance of 3 meters (the angle of the run-up was not specified so as to not interrupt the subjects natural kicking technique [6]); whilst completing an instep kick performed at maximum power. All three kicks were required to be taken with the preferred foot only.

This was then followed by the group-appropriate intervention:

- For lumbar SI, the lumbar roll technique was used as described by Szaraz [37].
- For the sacroiliac manipulation, a side lying technique was used with pisiform, posterior superior iliac spine contact as described by Bergmann, Peterson, and Lawrence [34].
- A combination of the above for the combination group.
- Laser intervention for the sham group.

Manipulation of a dysfunctional joint was considered successful if on reassessment after the manipulation, the motion palpation of that joint [33,34] showed improvement post manipulation and there was agreement between the researcher and the clinical supervisor (blinded to manipulation).

After the intervention the post-intervention measures were administered immediately (in order of lumbar and sacroiliac range of motion, repeated kicking outcomes and the subjective perception of the kick (improved, the same or worse)).

### Outcome measures

The range of motion was measured using a Saunders digital inclinometer [38]. Mayer, Kondraske, Beals and Gatchel [39], found that there was minimal error when using an inclinometer, however where error might be seen is on the examiners ability to locate bony anatomical landmarks (which was overcome in this study by marking the appropriate landmarks).

**Lumbar range of motion**: flexion, extension, lateral flexion and rotation motion was assessed according to the outlines provided in the manual by the Saunders Group [38] and Mayer, Kondraske, Beals and Gatchel [39].

**Sacroiliac Range of Motion (only flexion motion was assessed),** was assessed as outlined by Schafer and Faye [33], Bergmann, Peterson and Lawrence [34] and Saunders [38]. Calculations were done according to Arab et al., [40].

Performance was measured using the SpeedTrac™ Speed Sport Radar, which measured the kicking speed of the subjects. This device (EMG Companies, Wisconsin, USA) utilized Doppler signal processing to measure speeds of small projectiles. An internal antenna sends out radio waves at a specific frequency, so when a moving object, such as a kicked ball, enters the range of this signal it alters the frequency. The frequency of the reflected signal off the ball changes the frequency in proportion to the ball’s speed. The radar then displays the speed in the units of choice, in this case km/h. The signal transmitted is able to pass through netting without being affected. Therefore, a protective barrier can be placed between the moving object and the radar without affecting the accuracy of the measurements in any way. The speed range of the radar is 10-199 km/h, and the distance range is approximately nine meters. The accuracy of the radar is within 2-3 km/h [EMG Companies, Wisconsin, USA]. The SpeedTrac™ Speed Sport Radar was set up (specifically for this study), in the indoor arena behind the netting of the goal, so as to protect the unit; give the most accurate readings (7.5 meters away from the kicking point) and to give the subjects a target to assist aim.

In terms of the subjective outcomes of the study, subjects were all required to answer the following question post intervention, “Did you feel that your kicking speed increased or decreased or remained the same following the treatment?” (3 point Likert Scale).

## Statistics

SPSS version 15.0 was used to analyse the data. A *p* value < 0.05 indicated statistical significance. Demographic characteristics were compared between the groups using ANOVA tests. Intra-group comparisons of outcomes over time were achieved using within-subjects repeated measures ANOVA. A significant time effect indicated a significant effect of the intervention where each subject was used as their own control. Intra and inter-group comparison of interventions was achieved using between and within groups repeated measures ANOVA. A significant time verses group effect indicated that the interventions produced different results over time. Comparison of subjective and objective change in kicking speed was assessed using cross tabulations and Pearson’s chi square tests [41]. Normalcy of data were computed utilizing the Kolmogorov’s Smirnov test and normal probability plots.

## Results

**Figure 1:**
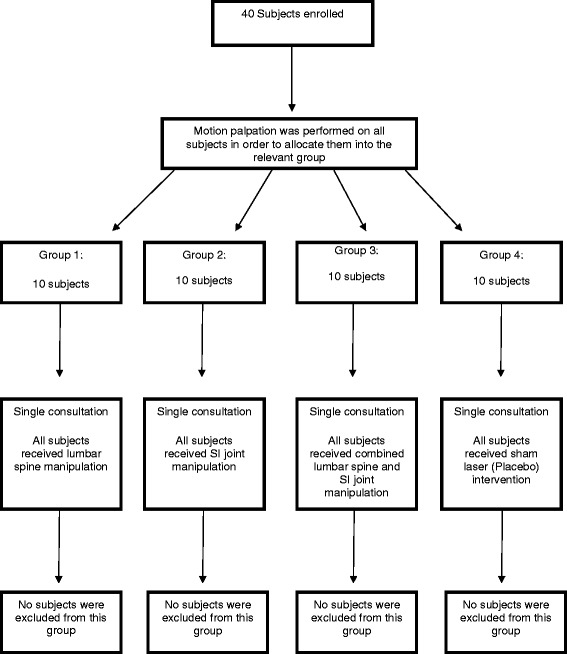
**Flow diagram showing subject intake and group allocation.**

Figure 1 outlines the flow of subjects through the study, based on the procedure outlined in the methodology. In terms of the baseline (pre-intervention) measurements between the groups there was no significant differences in terms of the subjects age, height and weight (demographic data) (Table 1).

**Table 1:** **Baseline measures between the groups**

	**Lumbar spine manipulation**		**SI joint manipulation**		**Combined**		**Sham**		***p*** **-value**
	**Mean**	**S.D**	**Mean**	**S.D**	**Mean**	**S.D**	**Mean**	**S.D**	
Height (cm)	175.3	4.3	175.6	6.6	180.6	8.5	177.3	3.9	0.209
Weight (kg)	75.6	5.6	75.8	11.1	81.9	8.0	75.3	7.1	0.234
Age (years)	23.5	3.4	24.1	4.0	23.1	3.4	23.0	2.9	0.890

There was no distinction of gender, all participants were male.

In terms of the pre-post intervention measures (using the Wilks Lambda tests), statistically significant increases in right and left lumbar spine rotation ranges of motion (Group 1) and lumbar spine extension, right and left lumbar spine rotation and sacroiliac joint flexion range of motion (Group3) were reflected. No significant changes were seen with Group 2 and Group 4 (Table 2).

**Table 2:** **The statistically significant ROM** ***p*** **values post intervention**

**Group**	**Intervention**	**Movement**	**Mean pre readings (degrees)**	**Mean post readings (degrees)**	***p*** **value**
**Group 1**	Lumbar spine manipulation	Lumbar Left Rotation	**4.95**	**5.33**	**0.026**
		Lumbar Right Rotation	**4.88**	**5.34**	**0.005**
		Lumbar Flexion	56.73	57.14	0.173
		Lumbar Extension	24.24	24.73	0.121
		Lumbar Right Lateral Flexion	25.45	25.73	0.107
		Lumbar Left Lateral Flexion	25.01	25.78	0.130
		Sacro-iliac motion (all)			>0.05
**Group 2**	SI joint manipulation	Lumbar Left Rotation	5.78	5.83	0.224
		Lumbar Right Rotation	5.88	5.98	0.343
		Lumbar Flexion	57.34	57.34	No change
		Lumbar Extension	24.83	24.93	0.343
		Lumbar Right Lateral Flexion	26.69	26.69	No change
		Lumbar Left Lateral Flexion	26.61	26.61	No change
		Sacro-iliac motion (all)			>0.05
**Group 3**	Lumbar spine and SI joint manipulation	Lumbar Extension	**24.6**	**25.81**	**0.014**
		Lumbar Flexion	56.49	56.69	0.162
		Lumbar Left Rotation	**4.89**	**5.59**	**0.001**
		Lumbar Right Rotation	**4.95**	**5.62**	**0.005**
		Lumbar Right Lateral Flexion	25.9	26.11	0.094
		Lumbar Left Lateral Flexion	25.61	26.13	0.125
		Right SI joint flexion	**7.305**	**7.98**	**0.024**
		All other SI motion			>0.05
**Group 4**	Sham laser	Lumbar Extension	24.7	24.7	No change
		Lumbar Flexion	58.68	58.68	No change
		Lumbar Left Rotation	5.26	5.49	0.269
		Lumbar Right Rotation	5.43	5.57	0.150
		Lumbar Right Lateral Flexion	26.24	26.24	No change
		Lumbar Left Lateral Flexion	26.16	26.16	No change
		Sacro-iliac motion (all)			>0.05

Analysis: Wilk’s lambda, with a 95% confidence interval.

In the intergroup comparisons Table 3 reflects the outcomes between the groups. This Table agrees with the outcomes of Table 2, where the significant differences seen pre-post for the individual groups are also the same reasons for the significant differences between the groups. Additionally all three manipulative Groups showed statistically significant increases in kicking speeds (Table 4) with the sham laser intervention no effect post intervention for kicking speed. Further, a significant relationship between perception of improved performance and the improvement in kicking speed was noted (Table 5 (average kicking speed)/Table 6 (maximum kicking speed)), but there was no correlation between in the improved kicking speed and range of motion of either the lumbar spine or the sacroiliac joints.

**Table 3:** **Intergroup comparisons**

	**Effect**	**Statistic**	***p value***
Inter-group Lumbar Flexion Comparison	Time	0.785	**0.015**
	Time*Group	0.782	0.183
	Group	0.515	0.675
Inter-group Lumbar Extension Comparison	Time	0.603	**<0.001**
	Time*Group	0.571	**0.003**
	Group	0.291	0.832
Inter-group Lumbar Left Lateral Flexion Comparison	Time	0.804	**0.022**
	Time*Group	0.757	0.124
	Group	0.588	0.627
Inter-group Lumbar Right Lateral Flexion Comparison	Time	0.733	**0.004**
	Time*Group	0.714	0.059
	Group	0.453	0.717
Inter-group Lumbar Left Rotation Comparison	Time	0.421	**<0.001**
	Time*Group	0.518	**0.001**
	Group	1.918	0.144
Inter-group Lumbar Right Rotation Comparison	Time	0.458	**<0.001**
	Time*Group	0.633	**0.012**
	Group	2.688	0.061
Inter-group Left SI Flexion 1 Comparison	Time	0.810	**0.025**
	Time*Group	0.768	0.148
	Group	0.269	0.847
Inter-group Left SI Flexion 2 Comparison	Time	0.862	**0.022**
	Time*Group	0.799	**0.042**
	Group	0.231	0.874
Inter-group Right SI Flexion 1 Comparison	Time	0.777	**0.012**
	Time*Group	0.729	0.078
	Group	0.941	0.431
Inter-group Right SI Flexion 2 Comparison	Time	0.760	**0.008**
	Time*Group	0.652	**0.017**
	Group	0.677	0.572
Inter-group average kicking speed comparison	Time	0.417	**<0.001**
	Time*Group	0.485	**<0.001**
	Group	0.349	0.790
Inter-group maximum kicking speed comparison	Time	0.592	<0.001
	Time*Group	0.586	< 0.001
	Group	0.330	0.804

Analysis: Wilk’s lambda, with a 95% confidence interval.

**Table 4:** **The statistically significant kicking speed** ***p*** **values post intervention**

**Group**	**Intervention**	**Average/maximum**	**Mean pre value**	**Mean post value**	**Average change**	***p*** **value**
**Group 1**	Lumbar spine manipulation	Average	93.67	97.19	3.52 km/h	0.009
		Maximum	97.2	100.9	3.70 km/h	0.029
**Group 2**	SI joint manipulation	Average	94.19	99.62	5.43 km/h	0.001
		Maximum	97	103.5	6.50 km/h	0.001
**Group 3**	Lumbar spine and SI joint manipulation	Average	96.03	102.6	6.57 km/h	< 0.001
		Maximum	101	105.6	4.60 km/h	0.002
**Group 4**	Sham	Average	100.02	98.58	-1.44 km/h	0.070
		Maximum	102.5	100.4	-2.10 km/h	0.096

Analysis: Wilk’s lambda, with a 95% confidence interval.

**Table 5:** **Cross tabulation of subjective change and objective change in average kicking speed**

			**Objective change in kicking speed (avg.)**		
			**Decrease**	**Same**	**Increase**
Subjective change in kicking speed	Decrease	Count	1	0	0
		Percentage	100.0%	.0%	.0%
	Same	Count	6	2	6
		Percentage	42.9%	14.3%	42.9%
	Increase	Count	1	0	24
		Percentage	4.0%	.0%	96.0%
Total		Count	8	2	30
*p* = 0.001		Percentage	20.0%	5.0%	75.0%

**Table 6:** **Cross tabulation of subjective change and objective change in maximum kicking speed**

			**Objective change in kicking speed (max)**		
			**Decrease**	**Same**	**Increase**
Subjective change in kicking speed	Decrease	Count	1	0	0
		Percentage	100.0%	.0%	.0%
	Same	Count	7	0	7
		Percentage	50.0%	.0%	50.0%
	Increase	Count	1	1	23
		Percentage	4.0%	4.0%	92.0%
Total		Count	9	1	30
*p* = 0.005		Percentage	22.5%	2.5%	75.0%

## Discussion

Due to the fact that in all four Groups the subjects were aware that they were being studied, it was considered that the full effects of the Hawthorne principles were negated as each group would have had a similar exposure to these effects and thus they would have been negated in the inter-group comparisons [32,42].

In light of the above, the results seem to suggest that, manipulation of the lumbar spine alone or in conjunction with the sacroiliac joint, seems to result in the most significant results in soccer players, with regards to kicking speed. This outcome may be attributed to the nature of the lumbar spine manipulation (rotation) used, coupled with slight extension [29], which lends itself to the recorded results where the only statistically significant differences were noted in the rotation and extension motions during inter-group comparisons (Table 2).

Additionally, the lumbar spine and sacroiliac joint combination manipulation group achieved the highest rate of improvement followed by the sacroiliac joint manipulation and then lumbar spine manipulation groups. This outcome concurs with the results obtained by Sood [43], where it was found that combination groups (thoracic and lumbar manipulation) resulted in the greatest degree of improvement and significant (p < 0.000) improvement for action cricket fast bowlers’ bowling speed. This is however in contrast to the findings of Le Roux [44] in amateur golfers where no significant improvements were seen in participants that received combination manipulation interventions. The difference may lie in the fact that Sood [43] and this study utilized athletes specialized roles as opposed to the amateur athletes in the study by Le Roux [44]. This is supported by Gowan et al. [45], Shrier et al. [46] and Lauro and Mouch [47].

It may however also need to be considered that athletes respond differently to manipulation when combined with another modality, as found in the study by Costa et al. [48], where a combination of manipulation and stretching improved the overall outcome for the athletes. This concept of muscle stretch may have adversely affected the outcomes of this study as athletes were placed in the lumbar roll position for the sacro-iliac and lumbar spine manipulation procedures and not for the sham laser intervention. This would have predisposed the intervention groups to muscle stimulation that may not have been present in the sham laser intervention group. Further, different responses to manipulation may be sport specific, position specific or perception specific in terms of the athlete, but may also be related to the definition of the musculoskeletal dysfunction and the intervention combinations/chiropractic care utilized [49], as well as the known neurophysiological effects of manipulation [50].

In this study, the performance results can only be due to the fact that athletes responded biomechanically (only range of motion was measured) to manipulation due to the effect of the manipulation on the joints and surrounding anatomical structures [26]. These outcomes therefore support and suggest that the biomechanical [2] role of the lumbar spine and sacroiliac joint manipulation does affect the instep kicking technique, through mechanisms suggested by Herzog, [23] and Pickar, [26]. Additionally, this concurs with results found in sports related research [30,51,52], indicating that increased movement of the biomechanical chain could increase the ball speed following foot-ball impact. Studies show that manipulative interventions (with controlled external conditions) resulted in players acquiring appropriate balance [2] between the musculoskeletal structures [20] leading to the improvements in performance.

Although the neurological effect of the manipulation was not measured in this study, it may have played a role in attaining the positive outcomes (increased kicking performance and increased range of motion). This possibility is supported by Pickar [26], Murphy [27]; Herzog [28], Symons [29] and Suter et al. [30], whose collective literature suggests that the outcome of a complex motion is most likely related to improved neurological co-ordination. This would suggest that an increased limb swinging speed and thus resultant kicking speed would result in improved performance.

The majority of the subjects’ perception was that the kicking speed had increased following the intervention. The perception of increase was matched with 96% of the subjects actually increasing the average kicking speeds and 92% increasing the maximum kicking speeds post intervention. There was, therefore, a statistically significant association between changes in kicking speeds immediately post intervention and the subjects’ perception of change in kicking speed.

## Limitations

One of the major limitations in this study was that of sample size.

## Future research

Future research needs to measure the neurological effect of manipulation and its impact in all forms of sport, but particularly soccer players to substantiate the outcomes of this study. Outcomes measures should include of measures neurological function, as it has been shown that manipulation results in neurological change in the cervical spine [52,53], which may also impact on biomechanical outcomes achieved.

Further research could also explore the effects of ipsilateral and contra lateral manipulation of the lumbar spine in combination with sacroiliac joint manipulation and how this would alter outcomes on kicking speed. Also, research on the effect of manipulation of the joints both lower down and higher up the kinematic chain be considered – either in isolation or in combination. Both of the above studies would benefit from utilizing professional players that have position specific training, which may increase the ability to detect smaller variances in range of motion and other outcome measures, as their kicking performance would be more consistent.

## Conclusions

This pilot study has demonstrated that lumbar spine and SI joint manipulation, when combined are an effective intervention for a short-term increase in kicking speed after one intervention. These outcomes are however only generalizable to those subjects that had improved motion of the dysfunctional motion segment on motion palpation after manipulation. Additionally, the use of a larger sample calculated on an *a priori* analysis would assist in validating the outcomes of this study and reduce the risk of type II error. This along with improved measures, obtained by utilizing a blinded assessor for the outcome measures; increase frequency of the intervention may assist in conclusively supporting or refuting the results obtained in this study.
